# Inorganic Contaminants in Rapadura from Latin America

**DOI:** 10.3390/foods14193285

**Published:** 2025-09-23

**Authors:** Raquel Fernanda Milani, Juliana Lopes Rodrigues, Sandra Julieth Henao Toro, Adriana Aparecida Mauri, Adriana Pavesi Arisseto Bragotto, Marcelo Antonio Morgano

**Affiliations:** 1 Institute of Food Technology, P.O. Box 139, Campinas 13070-178, SP, Brazil; julianalopesrdg@gmail.com (J.L.R.); drimauri@ital.sp.gov.br (A.A.M.); morgano@ital.sp.gov.br (M.A.M.); 2 Department of Food Science and Nutrition, School of Food Engineering, Universidade Estadual de Campinas—UNICAMP, Campinas 13083-862, SP, Brazil; sanheto@hotmail.com (S.J.H.T.); pavesi@unicamp.br (A.P.A.B.)

**Keywords:** non-centrifugal cane sugar, arsenic, cadmium, lead, risk assessment, ICP OES

## Abstract

Non-centrifugal cane sugar (NCS) is an artisanal product, also known as rapadura or brown sugar, and it is consumed both as a dessert and as a substitute to refined sugar. Despite being a source of essential nutrients, inorganic contaminants may be found in rapadura composition. Thus, this study aimed to optimize and to apply a method for As, Cd, and Pb determination in 72 NCS samples commercialized in Latin America. The method consisted of acid extraction of the inorganic contaminants using an ultrasound bath, and the determination was conducted by inductively coupled plasma optical emission spectroscopy (ICP OES). The method optimization was performed using a 2^2^ central composite design, considering time and oxidant mixture as key parameters, and the best conditions were verified by extracting the inorganic contaminants using a 15% oxidant mixture for 20 min. The acid extraction method using an ultrasound bath was considered adequate, with values for limits of detection and quantification between 0.005 and 0.039 mg kg^−1^, respectively, and trueness (spiked experiments and certified reference material) ranging from 93 to 108% for all analytes. Rapadura samples from Latin America presented low levels for As and Cd, <0.012 µg kg^−1^ and <0.005–0.045 mg kg^−1^, respectively. For Pb, all samples presented quantifiable levels, and 33% were not within the requirements established by the Brazilian and The Southern Common Market (MERCOSUR) regulations. Thus, monitoring the levels of inorganic contaminants in non-centrifugal cane sugar is fundamental to provide safety for consumers.

## 1. Introduction

Non-centrifugal cane sugar (NCS) is a solid product that can be made from sugarcane juice (*Saccharum officinarum* L.) after it has been evaporated, concentrated, and crystallized to a concentration greater than 90°Brix, and it may contain significant amounts of minerals, vitamins, and phenolic compounds, among other ingredients [1,2]. Most regions of the world consume NCS as a sweetener, with it being produced mainly in Latin America, the Caribbean, Asia, and Africa, and it is known as *jaggery* e *gur* (South Asia), *panela* (Colombia, Ecuador), *muscovado* (Philippines), *rapadura* (Brazil), and *kokuto* (Japan) [3].

Both the variety of sugarcane and the processing conditions have an impact on the composition of the NCS, whose production is mostly manual and depends on the region of production. The common process consists of cleaning and removing fibers, grinding sugarcane to obtain sugarcane juice, filtering/clarifying the juice, concentrating the juice with temperature (up to 90 °Brix), and solidifying molds [4]. The clarification process involves heating sugarcane juice to remove impurities like leaves, soil, and soluble solids. To completely clean the product, heating is combined with the action of certain compounds, such as natural binders (maceration of tree bark) or artificial ones, like polyacrylamide [5,6].

International Sugar Organization data show that NCS production is growing in Latin America, with production of around 177 million tons in 2021. Brazil is the largest raw sugar producer in the world with 20%, followed by India (19%), China (6%), the United States of America (5%), and Thailand and Mexico (4%) [7]. NCS consumption is common among the most insecure populations, particularly children, as it offers inexpensive carbohydrates [3]. According to Jaffe [1], eating this food can provide up to 37% and 20% of the recommended daily intake of copper and manganese minerals, respectively. Cane broths contain between 3.0 and 4.5% salts of organic and inorganic acids, whose formation occurs mainly during the grinding stages of sugarcane or by microbial activity. Besides these salts, unrefined sugars contain micronutrients, such as minerals and vitamins. Lee et al. [8] studied the mineral levels in refined and unrefined sugar samples purchased in South Korea, Thailand, Japan, and China and the authors reported high Ca, Fe, K, and Mg levels in unrefined sugars: for example, Ca ranged from 19.95 (refined white sugar) to 2732.21 mg kg^−1^ (unrefined sugar).

It is known that certain elements are not essential and can be harmful to human health. Arsenic (As), cadmium (Cd), and lead (Pb) are among the inorganic contaminants that are toxic and can damage the central nervous system, skin, liver, and kidneys [9]. Diet is considered the main source of inorganic contaminant exposure to humans. As, Cd, and Pb occur in plants mostly due to soil contamination, environmental factors, or even processing and transportation [10]. The main way food is exposed to arsenic is through water used in agriculture, which is found naturally in volcanic and mining regions. The presence of cadmium in food may be due to air contamination (urban areas), waste from industries, and the use of fertilizers in plantations [11,12]. Lead is a common indicator of environmental contamination and can be disseminated in the air through industrial activity, fertilizer use, or mining activities [10].

The occurrence of inorganic contaminants in sugarcane and its food products is frequently associated with both the environmental conditions (irrigation water, cultivars soil, use of fertilizers and pesticides) and the industrial processing (such as chemical residues from bleaching) [11,13]. Brazilian [14] and The Southern Common Market—MERCOSUR [15] regulations established maximum limits for As (0.10 mg kg^−1^) and Pb (0.10 mg kg^−1^) in sugar, whilst European regulations [16] established the Pb maximum limit (0.10 mg kg^−1^) in honey.

Few studies are reported in the literature concerning the occurrence of inorganic contaminants in NCS. Waheed et al. [17] investigated the levels of elements such as As, Br, Cd, Hg, Pb, Sb, and Se in 15 sugarcane processing samples, which included NCS. The authors used acid digestion with HNO_3_ and HClO_4_ as a method of sample preparation, and the analyses were conducted using techniques such as atomic absorption spectrometry (AAS) and neutron activation analysis (INAA). The NCS samples exhibited a wide range of element concentration levels, ranging from below the limit of detection (Cd) up to 129 μg kg^−1^ (Pb). Dos Santos et al. [18] reported a method for direct analysis of Cr, Cd, and Pb in rapadura by graphite furnace atomic absorption spectrometry (GF AAS). According to the proposed method, limits of quantification were between 4.5 and 49.3 μg kg^−1^, recoveries ranged between 95 and 103%, and precision (repeatability, coefficient of variation) was below 10% for all elements. Four rapadura samples from Brazil were analyzed and the values ranged from <32.8 to 160, <49.3 to 211.0, and <4.5 to 7.0 μg kg^−1^ for Cr, Pb, and Cd, respectively. Inductively coupled plasma optical emission spectroscopy (ICP OES) has also been used for the determination of inorganic contaminants in sugar [19,20,21,22,23]. This technique stands out due to its high detectability (with limits of detection in the order of μg L^−1^), the possibility of simultaneous measurements, as well as its shorter analysis time and lower cost compared to inductively coupled plasma mass spectrometry (ICP-MS) and INAA techniques.

Considering the lack of information on the presence of inorganic contaminants in rapadura, this study aims to optimize a simple, fast, and low-cost chemical consumption method to evaluate the occurrence of inorganic contaminants As, Cd, and Pb in rapadura samples from Latin American (Brazil, Colombia, Ecuador, Mexico, and Peru). Ultrasound-assisted acid digestion conditions were optimized, and the inorganic contaminant levels were monitored by ICP OES. Furthermore, risk assessment tools were employed to verify the potential health risks associated with the consumption of rapadura.

## 2. Materials and Methods

### 2.1. Samples

Samples were acquired from local markets (Table 1) and considered the main brands and presentation forms (block or granulated) commercialized in Brazil (*n* = 16), Colombia (*n* = 36), Ecuador (*n* = 12), Mexico (*n* = 3), and Peru (*n* = 5). The samples were homogenized using an inox grater and were kept under refrigeration until analysis.

### 2.2. Reagents and Solutions

Reagents used in all experiments were analytical grade or superior. Nitric acid and water were purified using a reverse osmosis system (Gehaka, São Paulo, Brazil) and a sub-boiling distiller (Berghof, Eningen, Germany), respectively. Hydrogen peroxide 30% (*w*/*v*) and hydrochloride acid 37% (*v*/*v*) from Merck (Darmstadt, Germany) were also employed. Analytical curves were prepared daily by successive dilutions of 1000 mg L^−1^ As and 100 mg L^−1^ multielemental standard solutions (Quimlab—Specsol, Jacarei, Brazil), ranging from 0.001 to 0.100 mg L^−1^ in HCl 5% (*v*/*v*).

### 2.3. Determination of Inorganic Contaminants in Rapadura

#### 2.3.1. Optimization of the Extraction Method

The optimization of the extraction method for inorganic contaminants using an ultrasound bath employed a 2^2^ central composite design of experiments considering the main parameters of ultrasound acid digestion: oxidant mixture (nitric acid + hydrogen peroxide, in percentage) and time (minutes) [24]. The water bath temperature was kept at 45 °C in order to preserve the volatile contaminants (As). To provide analytical signals, samples were spiked with 0.020 mg L^−1^ of As, Cd, and Pb and the recovery (response) was expressed as a percentage ratio between the value obtained and the expected value. The conditions and responses are described in detail in Table 2.

#### 2.3.2. Inorganic Contaminants in Rapadura by ICP OES

The samples were mineralized using an ultrasound bath (Easy 180H, Elma, Singen, Germany). Briefly, 1.0 g of rapadura samples were weighed in a graduate tube and solubilized with 7.5 mL of purified water. To this solution, 1.5 mL of purified nitric acid and 0.75 mL of hydrogen peroxide were added, and the tubes were transferred to an ultrasound bath at 45 °C for 20 min. At room temperature, the tubes were opened, and the volume was adjusted to 15 mL using purified water. Inorganic contaminants were determined by ICP OES (5100 VDV, Agilent Technologies, Tokyo, Japan) under the following conditions [25]: RF power = 1.35 kW, Ar flow rate = 12.0 L min^−1^, Ar auxiliary flow rate = 0.50 L min^−1^, seaspray nebulizer flow rate = 0.55 L min^−1^, double-pass spray chamber, axial plasma view, and wavelengths (nm): As, 193.696; Cd, 214.439; Pb, 220.353.

### 2.4. Quality Control and Statistical Analysis

For quality control, inorganic contaminant analysis was performed in triplicate, blank experiments following the same procedure used for rapadura samples and the analytical methods were validated considering The National Institute of Metrology, Standardization, and Industrial Quality—INMETRO recommendations [26], EURACHEM guidelines [27], and European Union—EU [28] terminology. Limits of detection (LOD) and quantification (LOQ) were calculated as described in Equations (1) and (2), being “s” = standard deviation of seven blank experiments and t = 3.143 (99% confidence level).

(1) LOD = 0 + t_(6, 0.01)_ × s

(2) LOQ = 10 × s

Linearity was verified by correlation coefficients (r), with r > 0.9995. Trueness was evaluated using spike experiments in four levels (0.010, 0.020, 0.050, and 0.100 mg L^−1^), according to the linear range of the analytical curve and a certified reference material of tea leaves (INCT-TL-1 Tea leaves, Instytut Chemii i Techniki Jądrowej, Warszawa, Poland): As = 0.106 ± 0.021 mg kg^−1^, Cd = 0.0302 ± 0.0040 mg kg^−1^ and Pb = 1.78 ± 0.24 mg kg^−1^. For precision (repeatability), the coefficient of variation (CV, in percentage) of seven independent experiments was verified.

The design of experiments, Pareto chart of effects, surface (contour plot), one-way analysis of variance (ANOVA) and Tukey’s test were performed using Statistica software 7.0 (StatSoft, Tulsa, OK, USA), at 95% of the confidence level. Values below the limit of quantification (LOQ) were treated as zero. The purpose of this approach was to avoid overestimating exposure or contamination and to prevent the introduction of pseudo-values that are not based on actual measurements.

### 2.5. Exposure Assessment and Risk Characterization

For exposure assessment, the daily consumption was calculated as described in Equation (3), where: L = inorganic contaminant level in the sample (mg/g), DI = recommended daily intake (g), and bw = body weight of the adults (60 kg) or children (15 kg).

(3) EDI—Estimated Daily Intake (mg/kg bw) = (L × DI)/bw

For recommended daily intake, portions recommended by the manufacturers were considered: 20 and 40 g. To characterize the risk associated with exposure to As, Cd, and Pb, the results were compared to the available health-based guidance values established in the literature [9,29,30,31].

## 3. Results and Discussion

### 3.1. Optimization of an Ultrasound Acid Digestion Method for Inorganic Contaminants in Rapadura by ICP OES

**Figure 1:**
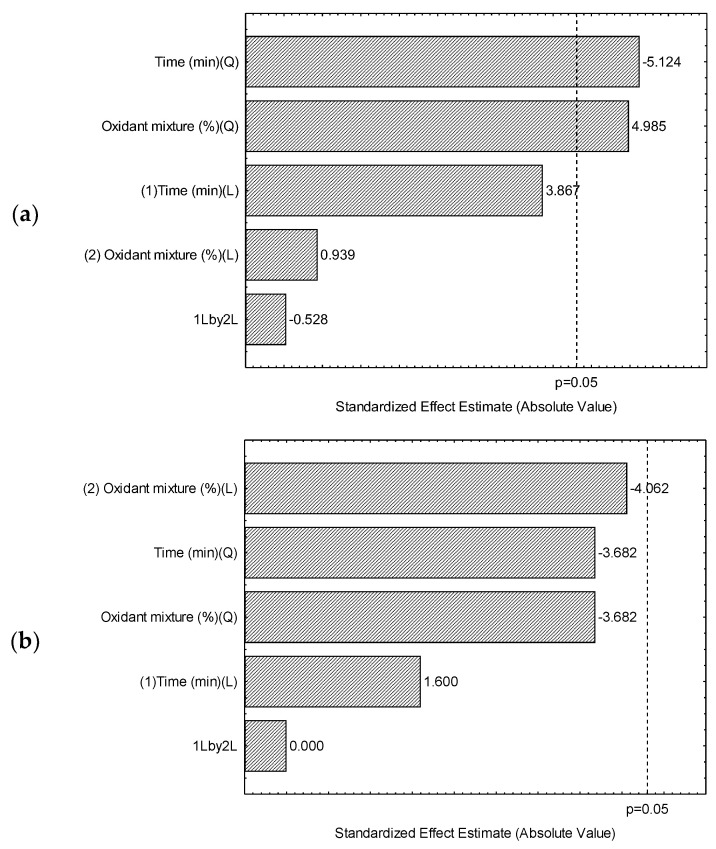

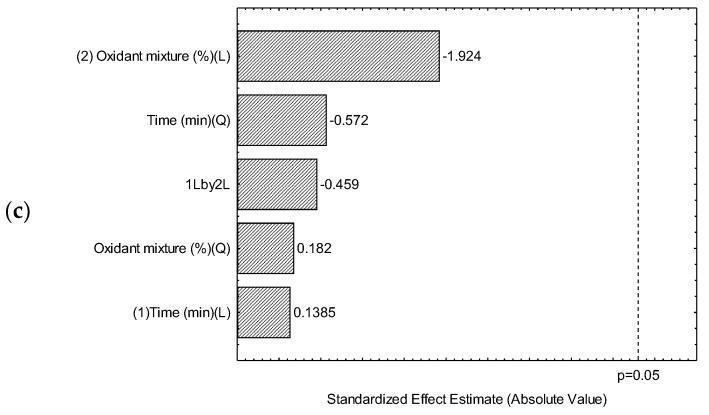
Pareto chart for recovery responses observed in 2^2^ central composite design of experiments: (**a**) Arsenic; (**b**) Cadmium; (**c**) Lead.

**Figure 2:**
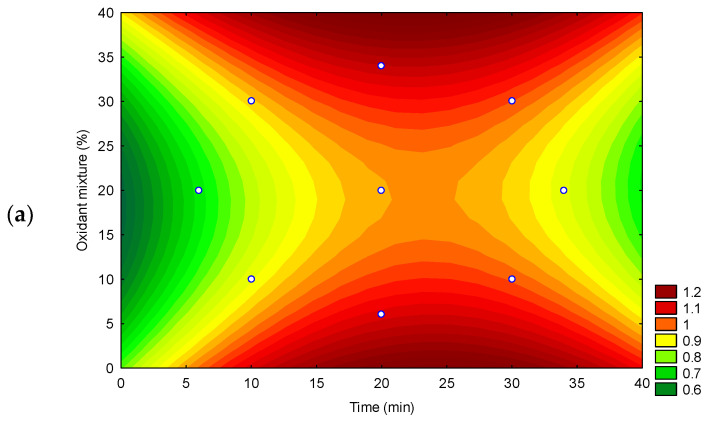

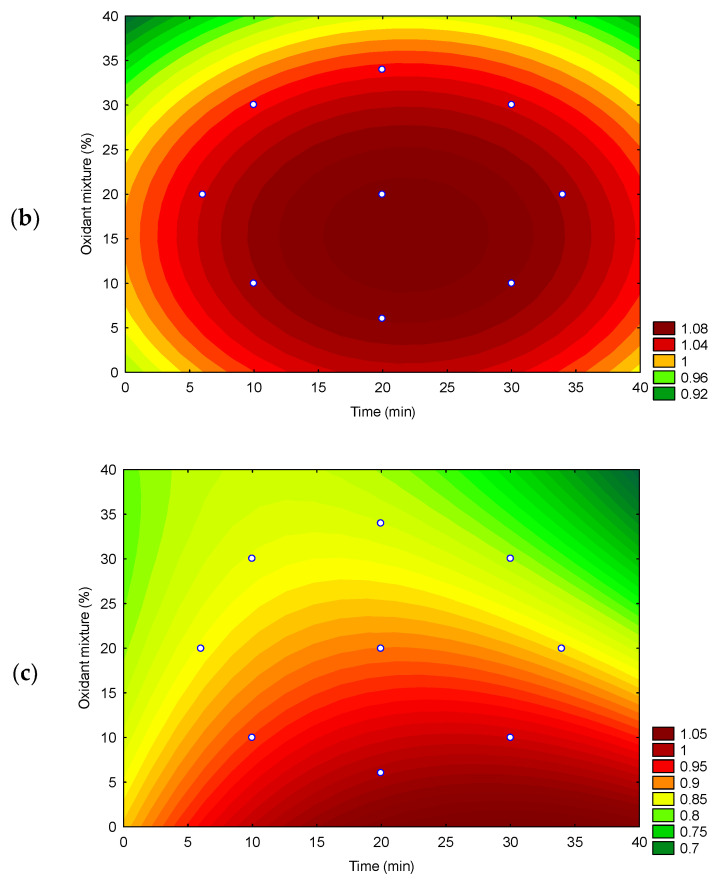
Surfaces observed for recovery responses in 2^2^ central composite design of experiments: (**a**) Arsenic; (**b**) Cadmium; (**c**) Lead.

A 2^2^ central composite design (CCD) was employed to optimize the method for analyzing As, Cd, and Pb in NCS by ultrasound digestion. The results are presented in Figure 1 and Figure 2.

The surfaces (contour plots) observed for recovery responses (Figure 2a–c) showed that the parameters time of extraction and mixture oxidant are significant, at a 95% confidence level, for the contaminant arsenic. By examining Figure 1a, it can be confirmed that the effects are inversely proportional: higher responses are observed when using higher percentages of oxidant mixture (positive effect) and shorter extraction times (negative effect). These results are consistent with Grindlay et al. [32], which reported the effect of high residual carbon levels on the increase in the analytical signal of arsenic by ICP OES.

The recovery values for Cd and Pb (Table 2) were between 80 and 110%, as recommended by INMETRO [26] and The Association of Official Analytical Collaboration (AOAC) International [33] for concentrations close to 0.100 ppm (mg kg^−1^). Nonetheless, for As some experiments showed recoveries that were not within the established range, which ranged from 71 to 117%. Thus, the surfaces were evaluated under a compromised condition of extraction time and oxidant mixture, which were defined as 20 min and 15% (oxidant mixture), respectively. The proposed method is suitable for the principles of ‘Green Chemistry’, which include the use of simple, low-consumption chemical reagents, diluted chemical reagent solutions, and reduced analysis time [34].

The method was validated and the figures of limit of detection, limit of quantification, trueness (recovery), and precision (repeatability) were evaluated, according to the INMETRO [26] and AOAC [33] recommendations. The results are shown in Table 3.

From Table 3, the limits of detection (LOD) and quantification (LOQ) ranged from 0.005 to 0.012 mg kg^−1^ and 0.015 to 0.039 mg kg^−1^, respectively [14,15,16]. These values were similar or better than those reported in the literature using ICP OES, GF-AAS, and FAAS. Dos Santos et al. [18] employed a direct method for detecting trace elements in brown sugar by GFAAS and reported LOQ values of 0.00447 and 0.0493 mg kg^−1^ for Cd and Pb, respectively. De Sousa et al. [35] studied a method for trace element determination in liquid aspartame sweeteners by ICP OES and obtained LOD values of 0.7 mg kg^−1^, 0.04 mg kg^−1^, and 0.9 mg kg^−1^ for As, Cd, and Pb, respectively. Alcívar et al. [11] studied Cd and Pb in sugarcane products from Ecuador by GF-AAS and reported LOQ values of 0.035 and 0.09 mg kg^−1^, respectively. Mekassa et al. [13] studied trace elements in white sugar samples from Ethiopia by FAAS and reported LOQ values of 0.021 and 0.007 mg kg^−1^ for Cd and Pb, respectively.

Recovery values ranged from 93 to 107% for spiked experiments and certified reference material, in agreement with INMETRO [26] and AOAC [33]: 80–110%. Precision (repeatability) was evaluated considering the coefficient of variation (CV, in percentage) and values were between 3 and 15% for As, Cd, and Pb.

### 3.2. Occurrence of Arsenic, Cadmium, and Lead in NCS Samples from Latin America

In general, low levels of As, Cd, and Pb were observed in NCS samples from Latin America (Brazil, Colombia, Ecuador, Mexico, and Peru). In Table 4 the results for the occurrence of inorganic contaminants in NCS samples are presented; the analysis was performed in triplicate, analytical blanks were performed using the same procedure, and values below the limit of quantification were treated as zero.

As shown in Table 4, all NCS samples acquired in Latin America presented non-detectable and non-quantifiable levels of total arsenic, whereas lead was detected and quantified in all NCS samples, ranging from 0.065 to 0.122 mg kg^−1^. For 33% of the NCS samples, Pb levels were found to be above the threshold established by Brazilian and MERCOSUR regulations for sugar (0.1 mg kg^−1^). These samples were produced in Brazil (Minas Gerais and Rio Grande do Sul), Ecuador (Quito), Mexico, and Peru (La Victoria and Lima).

Cadmium was found at detectable and quantifiable levels in 31% and 7% of the NCS samples, respectively, ranging from <0.015 (LOQ) to 0.045 μg kg^−1^. Although this inorganic contaminant is toxic and can cause alterations in the central nervous system [9], there are currently no limits established by the Brazilian and MERCOSUR regulations for this type of food [14,15]. The occurrence of cadmium and lead in NCS is often related to soil contamination by industrial waste, the use of fertilizers in plantations [10], environmental factors, or even due to NCS processing and transportation [9].

### 3.3. Exposure and Risk Assessment

To characterize the risk associated with inorganic contaminant (cadmium and lead) exposure, the highest exposure per portion (mg kg^−1^ bw day) was compared to the health-based guidance values (HBGV) established for each contaminant [9,29,30,31]. The exposure per portion was calculated for each NCS considering the portions recommended by the manufacturers (20 and 40 g portion) and the standard weights of 60 kg (adult) and 15 kg (child). For arsenic, all NCS samples acquired in Latin America presented non-quantifiable levels, and, therefore, the risk associated was not evaluated. The results are shown in Table 5.

In general, the percentage contribution of samples to the HBGV for the inorganic contaminants As, Cd, and Pb revealed safe values. The NCS sample from Peru had the highest Cd exposure, with estimates of 0.000120 and 0.000030 mg kg^−1^ bw for children and adults, respectively (Table 5). Considering a daily intake of 40 g of this NCS, the exposure values correspond to up to 4.8% and 1.2% of the tolerable weekly intake (TWI) for children and adults, respectively, as established by European Food Safety Authority—EFSA [12]. When considering the provisional tolerable monthly intake (PTMI) of 0.025 mg Cd kg^−1^ bw [9], the Cd contribution represented up to 0.5% and 0.1% of the PTMI for children and adults, respectively.

The highest exposure for Pb was found in an NCS sample from Mexico, with values calculated as 0.00033 and 0.00008 mg kg^−1^ bw for children and adults, respectively (Table 5). Considering a daily intake of 40 g of this NCS, the exposure values correspond to 2.8% and 0.5% of the benchmark dose lower confidence limit (BMDL) for children (BMDL_0.1_ = 0.012 mg Pb kg^−1^ bw day) and adults (BMDL_10_ = 0.015 mg kg^−1^ bw day), respectively [30]. Furthermore, the EFSA CONTAM panel established that Margins of Exposure (MOE) of 10 or greater would be sufficient for no appreciable risk of a clinically significant effect on Intelligence Quotient (IQ). All samples, whether for adults or children, had MOEs greater than 10, indicating that there is no significant risk of a clinically significant effect.

## 4. Conclusions

In this study, a reliable and simple method for determining inorganic contaminants As, Cd, and Pb in non-centrifugal cane sugar (NCS) was proposed. An ultrasound acid extraction method was optimized using a 2^2^ central composite design, and the determination was performed by ICP OES. The best conditions were verified by extracting the inorganic contaminants using a 15% oxidant mixture for 20 min at 45 °C. The limits of detection and quantification ranged between 5 and 39 µg kg^−1^ for all analytes, and the trueness was within the INMETRO and AOAC requirements (93 to 108%, for all analytes). Seventy-two NCS samples commercialized in Latin America were analyzed and the results revealed low levels for As (<12 µg kg^−1^) and Cd (<5–45 µg kg^−1^); Pb was quantifiable in all samples (65–122 µg kg^−1^) and was not in accordance with the requirements established by the Brazilian and MERCOSUR regulations for 33% of the NCS samples. Nevertheless, risk assessment revealed that a daily intake of 40 g of NCS could result in up to 2.8% and 0.5% of the benchmark dose lower confidence limit (BMDL) for Pb calculated for children and adults, respectively. Monitoring the levels of these inorganic contaminants is crucial in ensuring consumer safety since NCS is not the only source of these inorganic contaminants in the diet. Establishing maximum limits for NCS is necessary due to Pb levels being above the threshold of Brazilian and MERCOSUR regulations for sugar.

## Figures and Tables

**Table 1:** Composition of rapadura samples from Latin America.

Country	N	Origin
Brazil	16	Espírito Santo (*n* = 1), Minas Gerais (*n* = 13), and Rio Grande do Sul (*n* = 2).
Colombia	36	Boyacá (*n* = 1), Cundinamarca (*n* = 1), Nariño (*n* = 3), Santander (*n* = 1), and Valle del Cauca (*n* = 30)
Ecuador	12	Milagro (*n* = 2), Quito (*n* = 6), Vilcabamba (*n* = 1), and not available (*n* = 3).
Mexico	3	Not available (*n* = 3)
Peru	5	La Victoria (*n* = 1), Lima (*n* = 3), and not available (*n* = 1)

**Table 2:** Central composite design employed for optimization of ultrasound acid digestion method for inorganic contaminant determination in rapadura. Codified variables are presented between parentheses.

Experiment	Parameters		Response (Recovery, %)		
	Time (min)	Oxidant Mixture (%)	As	Cd	Pb
1	10 (−1)	10 (−1)	82	103	90
2	10 (−1)	30 (+1)	96	102	83
3	30 (+1)	10 (−1)	92	107	95
4	30 (+1)	30 (+1)	102	106	80
5	6 (−√2)	20 (0)	71	106	87
6	34 (+√2)	20 (0)	89	104	88
7	20 (0)	6 (−√2)	117	109	102
8	20 (0)	34 (+√2)	107	101	84
9	20 (0)	20 (0)	93	109	100
10	20 (0)	20 (0)	92	107	84
11	20 (0)	20 (0)	99	109	86

**Table 3:** Results for validation: limits of detection (LOD) and quantification (LOQ), precision (coefficient of variation, CV), and trueness (certified refence material and spiked tests in four levels of concentration).

Figure of Merit		Inorganic Contaminant		
		Arsenic	Cadmium	Lead
LOD (*n* = 7) (mg kg^−1^)		0.012	0.005	0.012
LOQ (*n* = 7) (mg kg^−1^)		0.037	0.015	0.039
Precision—repeatability (*n* = 7) (CV, %)		15	3	3
Spiked experiments (*n* = 3) (Trueness—recovery, %)	Level 1 (10 µg kg^−1^)	104 ± 3	108 ± 1	97 ± 5
	Level 2 (20 µg kg^−1^)	100 ± 7	105 ± 3	94 ± 4
	Level 3 (50 µg kg^−1^)	96 ± 2	104 ± 1	101 ± 1
	Level 4 (100 µg kg^−1^)	102 ± 1	107 ± 2	105 ± 1
CRM (*n* = 3)	Certified value (mg kg^−1^)	0.106 ± 0.021	0.0302 ± 0.0040	1.78 ± 0.24
	Obtained value (mg kg^−1^)	0.099 ± 0.011	0.028 ± 0.002	1.66 ± 0.48
	Recovery (%)	93 ± 10	93 ± 6	93 ± 3

Note: Limit of detection (LOD = 3.143 × s); limit of quantification (LOQ = 10 × s); CRM = certified reference material (INCT-TL-1 tea leaves).

**Table 4:** Occurrence of inorganic contaminants in rapadura from Latin America.

Country		Inorganic Contaminant (mg kg^−1^)		
		Arsenic	Cadmium	Lead
Brazil (*n* = 16)	Mean	<LOQ	<LOQ	0.085 ^ab^
	Range	<LOQ–<LOQ	<LOQ–<LOQ	0.065–0.118
Colombia (*n* = 36)	Mean	<LOQ	0.001 ^b^	0.074 ^b^
	Range	<LOQ–<LOQ	<LOQ–0.043	0.067–0.092
Ecuador (*n* = 12)	Mean	<LOQ	<LOQ	0.096 ^a^
	Range	<LOQ–<LOQ	<LOQ–<LOQ	0.078–0.108
Mexico (*n* = 3)	Mean	<LOQ	0.005 ^a^	0.101 ^a^
	Range	<LOQ–<LOQ	<LOQ–0.015	0.081–0.122
Peru (*n* = 5)	Mean	<LOQ	0.018 ^a^	0.107 ^a^
	Range	<LOQ–<LOQ	<LOQ–0.045	0.085–0.119

Note: LOQ (limit of quantification): As = 0.037 mg kg^−1^, Cd = 0.015 mg kg^−1^, and Pb = 0.039 mg kg^−1^; ^a,b^ Mean values between different columns with the same letter are not significantly different at *p* > 0.05, according to Tukey’s test.

**Table 5:** Results for exposure per portion of inorganic contaminants (mg kg^−1^ bw) and the contribution to the reference value and health-based guidance values (HBGV), considering two NCS consumption scenarios for children (body weight, bw = 15 kg) and adults (bw = 60 kg).

Inorganic Contaminant	Population Group	Highest Exposure, mg kg^−1^ bw day^−1^ (Contribution to the HBGV)		
		Portion: 20 g	Portion: 40 g	Sample Region
Cadmium	Children	0.000060 (0.2% PTMI; 2.4% TWI)	0.000120 (0.5% PTMI; 4.8% TWI)	Peru
	Adults	0.000015 (0.1% PTMI; 0.6% TWI)	0.000030 (0.1% PTMI; 1.2% TWI)	
Lead	Children	0.00016 (1.3% BMDL_0.1_)	0.00033 (2.8% BMDL_0.1_)	Mexico
	Adults	0.00004 (0.3% BMDL_10_)	0.00008 (0.5% BMDL_10_)	

Note: BMDL = benchmark dose lower limit; BMDL_0.1_ for Pb (children) = 0.012 mg kg^−1^ bw; BMDL_10_ for Pb (adults) = 0.015 mg kg^−1^ bw; PTMI = provisional tolerable monthly intake: Cd = 0.025 mg kg^−1^ bw; TWI = tolerable weekly intake: Cd = 0.0025 mg kg^−1^ bw [9,29,30,31].

## Data Availability

The original contributions presented in this study are included in the article. Further inquiries can be directed to the corresponding author.
